# Human bone marrow mesenchymal stem cells-derived microRNA-205-containing exosomes impede the progression of prostate cancer through suppression of RHPN2

**DOI:** 10.1186/s13046-019-1488-1

**Published:** 2019-12-17

**Authors:** Shuangjian Jiang, Chengqiang Mo, Shengjie Guo, Jintao Zhuang, Bin Huang, Xiaopeng Mao

**Affiliations:** 1 Department of Urology Surgery, the First Affiliated Hospital, Sun Yat-Sen University, No. 58, Zhongshan No. 2 Road, Guangzhou, 510080 Guangdong Province People’s Republic of China; 2 Department of Urology Surgery, Sun Yat-sen University Cancer Center, Guangzhou, 510060 People’s Republic of China; 3 Department of Urology Surgery, the Eastern Hospital of the First Affiliated Hospital, Sun Yat-Sen University, Guangzhou, 510700 People’s Republic of China

**Keywords:** Human bone marrow mesenchymal stem cells, Exosomes, MicroRNA-205, Prostate cancer, RHPN2

## Abstract

**Background:**

Human bone marrow mesenchymal stem cells (hBMSCs) are implicated in cancer initiation and metastasis, sometimes by releasing exosomes that mediate cell communication by delivering microRNAs (miRNAs). This study aimed to investigate the physiological mechanisms by which exosomal miR-205 derived from hBMSCs may modulate the growth of prostate cancer cells.

**Methods:**

Microarray-based gene expression profiling of prostate cancer was adopted to identify differentially expressed genes and regulatory miRNAs, which identified the candidates RHPN2 and miR-205 as the study focus. Then the binding affinity between miR-205 and RHPN2 was identified using in silico analysis and luciferase activity detection. Prostate cancer cells were co-cultured with exosomes derived from hBMSCs treated with either miR-205 mimic or miR-205 inhibitor. Subsequently, prostate cancer cell proliferation, invasion, migration, and apoptosis were detected in vitro. The effects of hBMSCs-miR-205 on tumor growth were investigated in vivo.

**Results:**

miR-205 was downregulated, while RHPN2 was upregulated in prostate cancer cells. RHPN2 was a target of miR-205, and upregulated miR-205 inhibited prostate cancer cell proliferation, invasion, and migration and promoted apoptosis by targeting RHPN2. Next, experiments demonstrated that hBMSCs-derived exosomes carrying miR-205 contributed to repressed prostate cancer cell proliferation, invasion, and migration and enhanced apoptosis. Furthermore, in vivo assays confirmed the inhibitory effects of hBMSCs-derived exosomal miR-205 on prostate cancer.

**Conclusion:**

The hBMSCs-derived exosomal miR-205 retards prostate cancer progression by inhibiting RHPN2, suggesting that miR-205 may present a predictor and potential therapeutic target for prostate cancer.

## Background

Prostate cancer is the most common malignancy among men and one of the most common causes of cancer related death among men worldwide [1]. It has been estimated that 1.6 million men are diagnosed with prostate cancer and 366,000 die from the cancer each year [2]. Several factors including age, race, family heritability, are considered to be associated with the development of prostate cancer [3]. Recent studies have reported that patients diagnosed with prostate cancer tend to be over-treated due to the lack of efficient predictive biomarkers [4]. Interestingly, the role of mesenchymal stem cells (MSCs) in cancer progression has attracted great attention in recent years [5]. Human mesenchymal stem cells (hMSCs) from adult bone marrow can be induced to differentiate into multiple mesenchymal tissues [6]. Moreover, increasing studies have proposed the contribution of bone marrow-derived mesenchymal stem cells (BMSCs) to tumor growth and metastasis [7]. Therefore, our research interests arose considering the possible mechanisms of the involvement of BMSCs in prostate cancer.

Stem cells can release exosomes, and stem cells-derived exosomes are often used experimentally as a potential therapeutic target for the paracrine actions of MSCs [8]. Exosomes are attractive targets for cancer treatment due to their small size (40 ~ 100 nm) and great impact on cancer cell physiology [9]. The exact effects of exosomes on the promotion or inhibition of prostate cancer have already been investigated [10]. Exosomes carrying proteins, messenger RNAs (mRNAs), and microRNAs (miRNAs) can be transferred between cells [11]. miRNAs are small non-coding RNA molecules that can regulate gene expression at the posttranscriptional level via degradation or repression of target miRNAs [12]. In addition, the exosomal miRNAs are now considered as biomarkers for prostate cancer [13, 14]. Moreover, exosome-mediated miRNA delivery has been extensively reported to contribute to prostate cancer chemoresistance [15]. In a previously reported study, miR-205 acts as a tumor suppressor gene with a distinct role in the progression of prostate cancer [16]. In the present study, the microarray-based analysis implicated rhophilin Rho GTPase binding protein 2 (RHPN2) in prostate cancer. RHPN2, which is located on chromosome 9q13.11, encodes members of the rhophilin family of Ras-homologous (Rho)-GTPase binding proteins [17]. Of note, RHPN2 has been reported to be involved in the development of malignant glioma [18]. In addition, the genetic variant of RHPN2 (rs10411210) gene has been suggested to be a prognostic biomarker for patients with surgically resected colorectal cancer [19]. However, the effect of human BMSCs (hBMSCs)-derived exosomes on the occurrence and progression of prostate cancer remain poorly understood. Therefore, in this study, we intend to explore the effects of hBMSCs-derived exosomal miR-205 on the progression of prostate cancer via regulating RHPN2 so as to provide novel potential therapeutic strategies for combating prostate cancer.

## Materials and methods

### Ethics statement

All participates enrolled in the study signed informed consent documentation. This study was approved by the Ethics Committee of the First Affiliated Hospital, Sun Yat-Sen University. The animal experiments were performed in strict accordance to the Guide for the Care and Use of Laboratory animals published by the US National Institutes of Health.

### Microarray-based analysis

The prostate cancer-related microarray profiles of GSE26910 and GSE30994 were obtained from the Gene Expression Omnibus (GEO) database (https://www.ncbi.nlm.nih.gov/geo/). GSE26910 was used to detect the gene expression profile of the stroma tissue around the six cases of primary infiltrative prostate cancer and six cases of normal prostate as reference material. GSE30994 was used to detect the gene expression profile of three cases of prostate cancer and three samples of normal prostate [20, 21]; we then compared the gene expression profile between normal prostate and prostate cancer samples using the Limma software package in R language to identify the differentially expressed genes (DEGs) associated with prostate cancer. We used |logFC| > 2, *p* value < 0.05 as the threshold, and employed the pheatmap package in R language to construct the heatmaps of DEGs. The expression of RHPN2 in The Cancer Genome Atlas (TCGA) database was analyzed using UALCAN database (http://ualcan.path.uab.edu/analysis.html). The DIANA database (http://diana.imis.athena-innovation.gr/DianaTools/index.php?r=microT_CDS/index), miRDB database (http://mirdb.org/miRDB/index.html), mirDIP database (http://ophid.utoronto.ca/mirDIP/index.jsp#r), miRSearch database (https://www.exiqon.com/miRSearch), and TargetScan database (http://www.targetscan.org/vert_71/) were employed to predict the miRNAs that might regulate RHPN2.

### Study subjects

Androgen-dependent LNCaP prostate cancer cell line was incubated in the Roswell Park Memorial Institute (RPMI) 1640 medium supplemented with 10% fetal bovine serum (FBS). BMSCs were cultured in Dulbecco’s modified Eagle’s medium (DMEM) supplemented with 10% FBS with 5% CO_2_ at 37 °C. BMSCs were extracted from the bone marrow of healthy adults and then purified. The reverse transcription quantitative polymerase chain reaction (RT-qPCR) was adopted to determine miR-205 expression in cells at passage 3 to screen out the cell line.

### Plasmid transfection

LNCaP cells were inoculated in 6-well plates at a density of 2 × 10^5^ cells/well 1 day before transfection. Plasmids were transduced when cell confluence reached 60–80%. LNCaP cells were treated with miR-205/negative control (NC) mimic, miR-205/NC inhibitor, short hairpin RNA (shRNA) targeting RHPN2/NC, and RHPN2/NC (Shanghai GenePharma Co., Ltd., Shanghai, China), respectively in accordance with the instructions of Lipofectamine 2000 (Invitrogen, Carlsbad, CA, USA). In order to reduce toxicity, the supernatant was replaced with fresh medium at 6 h after transfection.

### RT-qPCR

The total RNA was extracted using a Trizol Kit (Invitrogen, Carlsbad, California, USA), and the diethylpyrocarbonate (DEPC)-treated ultrapure water was used to dissolve RNA. The absorbance values of RNA at the wavelength of 260 nm and 280 nm were evaluated in the ND-1000 ultraviolet spectrophotometer (Nanodrop, Thermo Fisher Scientific Inc., Waltham, MA, USA) to identify the concentration and purity of the total extracted RNA. Next, the extracted RNA was reversely transcribed into complementary DNA (cDNA) following the instructions of the Reverse Transcription Kit (Fermentas Inc., Hanover, MD, USA). RT-qPCR was then conducted using the TaqMan probe method. The reaction system was performed according to the instructions of the kit (Fermentas Inc., Hanover, MD, USA). The primer sequences are shown in Table 1. The quantitative PCR instrument (Bio-Rad iQ5, Bio-Rad, Richmond, Cal., USA) was employed to conduct RT-qPCR. U6 was regarded as the internal reference of miR-205, while glyceraldehyde-3-phosphate dehydrogenase (GAPDH) was the internal reference of RHPN2. The ratio of relative gene expressions was analyzed by 2^-ΔΔCt^ method. The experiment was repeated three times independently [22].

**Table 1:** Primer sequences for RT-qPCR

Gene	Sequence (5′-3′)
miR-205	Forward: 5′-GGAGTTTAAGTTGTGTATGGAAGTG-3′
	Reverse: 5′-AAAACAAATATTTCTTTTATAATCCAAA-3′
U6	Forward: 5′-GCTTCGGCAGCACATATACTAAAAT-3′
	Reverse: 5′-CGCTTCACGAATTTGCGTGTCAT-3′
RHPN2	Forward: 5′-AGAACGACGGCTACTTTCGG-3′
	Reverse: 5′-CACGGCTTTCAGGATCTGC-3′
GAPDH	Forward: 5′-ATGGAGAAGGCTGGGGCTC-3′
	Reverse: 5′-AAGTTGTCATGGATGACCTTG-3′

Note: *RT-qPCR* Reverse transcription quantitative polymerase chain reaction, *miR-205* MicroRNA-205, *RHPN2* Rhophilin Rho GTPase binding protein 2, *GAPDH* Glyceraldehyde-3-phosphate dehydrogenase

### Western blot analysis

The total protein was extracted, and protein concentration was quantified using a bicinchoninic acid (BCA) kit (Thermo Fisher Scientific, Rockford, IL, USA). Then, 30 μg protein samples were treated with sodium dodecyl sulfate-polyacrylamide gel electrophoresis (SDS-PAGE) gel and subsequently transferred to a polyvinylidene fluoride (PVDF) membrane (Amersham plc, GE Healthcare, Chicago, Illinois, USA). After blocked in bovine serum albumin (BSA) at room temperature for 1 h, the membrane was incubated with primary antibodies of CD63 (1: 1000, ab134045), Hsp70 (1:1000, ab79852, Abcam, UK), Calnexin (1:1000, ab22595, Abcam, UK), matrix metalloproteinase (MMP)-2 (1:1000, ab37150, Abcam, UK), MMP-9 (1:1000, ab73734), Ki67 (1:500, ab15580), proliferating cell nuclear antigen (PCNA) (1:1000, ab18197), B-cell lymphoma 2 (Bcl-2) (1:1000, ab196495), Bcl-2-associated X protein (Bax) (1:500, ab53154), GAPDH (1:5000, ab37168, Abcam, UK) and mouse antibody against RHPN2 (1:1000, H00085415-B01P, Bio-Techne China Co., Ltd., Shanghai, China), at 4 °C overnight. All the above antibodies were purchased from Abcam Inc. (Cambridge, MA, USA). After washing with phosphate-buffered saline (PBS) containing 0.1% Tween-20 (PBST) for three times, 10 min each time, the membrane was subjected to another incubation with secondary goat anti-rabbit or anti-mouse (1: 10000, Jackson Immunoresearch Laboratories, West Grove, PA, USA) at 37 °C for 1 h. The samples were then washed with PBST for 3 times and developed using an optical luminescence instrument (General Electric Co., Erie, PA, USA). The band intensities were subsequently assessed using the Image Pro Plus 6.0 software (Media Cybernetics, Silver Spring, USA). The relative protein expression was finally determined. The experiment was repeated three times independently.

### 5-ethynyl-2′-deoxyuridine (EdU) assay

The cell culture plate was added with medium containing EdU (1000: 1), followed by incubation at room temperature for 2 h, and washing with PBS. Subsequently, the cells were fixed in 40 g/L paraformaldehyde for 30 min, followed by incubation with glycine for 8 min and washing with PBS containing 0.5% Triton X-100. After that, each well was added with Apollo® and rinsed twice with methanol and then PBS, respectively. At last, the cells were incubated with Hoechst 3334 at room temperature without light exposure at room temperature for 20 min. Cells were observed under a fluorescence microscope with excitation at 550 nm, the red stained cells were proliferating cells, while under excitation at 350 nm, blue stained cells were surviving cells. A total of 3 visual fields were randomly selected under a microscope. Cells stained with EdU (proliferating cells) and with Hoechst 33342 (total cells) were counted. EdU proliferating rate (%) = the number of proliferating cells/the number of total cells × 100%. The experiment was repeated three times independently.

### Transwell assay

The Matrigel (YB356234, Shanghai YuBo Biological Technology Co., Ltd., Shanghai, China) was dissolved into liquid state at 4 °C overnight. Then, 200 μL serum-free medium was mixed with 200 μL Matrigel at 4 °C. Each well in the apical chamber was added with 50 μL Matrigel and incubated for 2–3 h so that the gel was changed to the solid state. Cells were detached, counted and prepared into cell suspension using medium containing 20% FBS. Each well in the apical chamber was added with 200 μL cell suspension, and the basolateral chamber was added with 800 μL conditioned medium containing 20% FBS. After incubation at 37 °C for 20–24 h, the Transwell plate was rinsed twice with PBS, and cells were fixed with formaldehyde for 10 min, and washed with running water for 3 times. Subsequently, the samples were stained with 0.1% crystal violet for 30 min at room temperature and washed twice with PBS. The cells adhering to the surface were wiped off using cotton balls. After 24 h, the cells were observed, photographed, and counted in at least 4 randomly selected fields under an inverted microscope. There was no need to spread the Matrigel in the Transwell migration assay where the incubation lasted for 16 h.

### Clonogenic assay

Cells were detached by 0.25% trypsin-ethylenediaminetetraacetic acid (EDTA) and a single cell suspension was collected. After counting, the cells were gradient diluted and 6-well plates were added with 1000 cells per well and shaken to distribute the cells evenly. Next, cells were incubated with 5% CO_2_ at 37 °C for 3–4 weeks. The obvious colonies formed in the culture medium suggested the end of incubation. The culture fluid was the removed, and cells were fixed in polyformic acid for 15 min and stained with crystal violet for 20 min, followed by elution with running water. Then, the number of colonies per well was counted.

### Flow cytometry

After transfection for 48 h, cells were washed with PBS for three times and centrifuged, followed by the removal of supernatant. After resuspension in PBS, cell concentration was adjusted to 1 × 10^5^ cells/mL. Next, the cells were added with pre-cooled 75% ethanol (1 mL), fixed at 4 °C for 1 h, and centrifuged to discard the ethanol. After being washed with PBS, cells were incubated with 100 μL RNase A avoiding exposure to light, and incubated in water bath at 37 °C for 30 min. Then, 400 μL propidium iodide (PI) (Sigma-Aldrich Chemical Company, St Louis, MO, USA) was added to the cells for incubation at 4 °C for 30 min. Flow cytometer (CytoFLEX, Beckman Coulter Inc., Chaska, MN, USA) was then utilized to record the red fluorescence at the excitation wavelength of 488 nm to detect cell cycle distribution.

After transfection for 48 h, cells were detached with trypsin without EDTA, collected into a flow tube, and centrifuged, with the supernatant removed. According to the instructions of the Annexin-V-fluorescein isothiocyanate (FITC) apoptosis detection kit (Sigma-Aldrich Chemical Company, St Louis, MO, USA), Annexin-V-FITC, PI, and HEPES were mixed at a ratio of 1: 2: 50. A total of 1 × 10^6^ cells were resuspended in 100 μL dye liquor, followed by incubation with 1 mL HEPES butter. Fluorescence was initiated by excitation at 488 nm and was measured by emission filters at 525 nm (FITC) and 620 nm (PI). The cell apoptosis was finally detected.

### Culture and identification of hBMSCs

Young or middle-aged patients with limb fracture and hip replacement at the Department of Orthopedics, the First Affiliated Hospital, Sun Yat-Sen University were invited to serve as bone marrow donors, with exclusion of patients with hematological diseases. When the broken end of fracture was cleaned during operation or reaming of femoral medullary cavity was conducted after interception of femoral head, fresh bone marrow (5–7 mL) was collected, and placed in a 10 mL sterile syringe preloaded with heparin sodium. Next, the samples were transferred into a 15 mL sterile centrifuge tube. A 25 cm^2^ sterile culture bottle was added with 6 mL DMEM/Ham’s F-12 medium (F12) containing 10% FBS. The fresh bone marrow blood sample (1 mL) was added into the culture bottle, which were labeled the name, batch, date, and other information. The bottles were then placed in an incubator with 5% CO_2_ at 37 °C. Samples from the same batch were usually cultured in 3–5 bottles to improve the success rate of culture. The passage culture was conducted. At 24, 48 and 72 h, the culture bottle with different numbers was replaced in full volume, and the uncoated cells such as red blood cells and adipocytes were discarded. The three different time points were selected to improve the success rate of cultivation. The culture bottle was added with 5 mL DMEM/F12 fresh medium containing 10% FBS. After 4–7 days, when the adherent BMSCs reached 80–90% confluence, cells were sub-cultured again. Purification and amplification of BMSCs were conducted at the third passage. The cell adherence and growth were observed under an inverted microscope. The flow cytometer was adopted to detect the surface markers of hBMSCs, CD34, CD45, CD14, CD105, CD90, CD29, and CD73.

### Lentivirus transduction

The transient co-transfection system was used for virus packaging and the plasmids (1 μg PMD2G, 3 μg PSPAX2, and 4 μg Prutou3-mChely*/*miR-205 (miR-NC/miR-205) were co-transfected into LNCaP cells in a 60 mm culture dish. After transfection for 24 h, the cell supernatant was collected and the cells were further incubated with fresh culture medium for 24 h before collecting. The two batches of supernatants were mixed and centrifuged to detect the virus titer. The target cells were transduced with the virus. The lentivirus-transduced hBMSCs were seeded into 24-well plates (5 × 10^4^ cells/well). Before transduction, infection complex medium containing 500 μL lentivirus supernatant, 500 μL fresh culture medium and 8 μg polyacrylamide (Sigma-Aldrich Chemical Company, St Louis, MO, USA) was used to replace the medium to assist the internalization of virus particles. The plates were then centrifuged at 2100 g at 37 °C for 1 h, and the medium was replaced with the fresh culture medium.

### Co-culture of hBMSCs and prostate cancer LNCaP cells

LNCaP cells and hBMSCs were detached by trypsin, centrifuged at 1000×g for 5 min, and resuspended in 3 mL DMEM. The suspension (1 mL) was diluted 20-fold, which was fully mixed, and 10 μL suspension was counted. The hBMSCs and LNCaP cells at passage 5 were co-cultured in 6-well plates in the Transwell apical and basolateral chambers. When conducting co-culture, DMEM containing 10% serum was used in the apical chambers and DMEM containing 15% serum was used in the basolateral chambers. Cells were co-cultured for 4–5 days and fed every 1–2 days. The cell culture media in both chambers were simultaneously replaced by fresh media. The cell density in the apical chambers was 20,000, while it in the basolateral chambers was 30,000. After 72 h, upon reaching more than 80% confluence, hBMSCs and LNCaP cells were collected and rinsed with PBS for the subsequent experiments.

Exosomes were then treated with inhibitor GW4869. In brief, when hBMSCs reached 80% confluence, GW4869 with a final concentration of 5 μM was added to the cells for 48 h of treatment. Prostate cancer cells were then subjected to further treatment with MSC medium with overexpressed miR-205 following with or without GW4869 treatment, which were used for subsequent experiments [23].

### Dual-luciferase reporter gene assay

The biological prediction website microRNA.org was used to verify whether RHPN2 was a target gene of miR-205, which was further verified by dual-luciferase reporter gene essay. The artificially synthesized RHPN2 3’untranslated region (3’UTR) gene fragment was introduced into a pMIR-reporter (Promega Corporation, Madison, WI, USA) through the endonuclease sites SpeI and Hind III. The complementary sequence mutation sites of seed sequences were designed on wild type (WT) of RHPN2 3’UTR. After restriction endonuclease digestion, the target fragment was inserted into the pMIR-reporter reporter plasmids via T4 DNA ligase. The WT and mutant (MUT) plasmids were co-transfected with miR-205 mimic and mimic NC into HEK-293 T cells (Beinuo Life Science, Shanghai, China). After transfection for 24 h, the Dual-Luciferase Reporter Assay System (Promega Corporation, Madison, WI, USA) was employed to detect luciferase activity. The experiment was repeated three times independently.

### Isolation of hBMSC exosomes

FBS was centrifuged for 18 h at 100000×g in advance to remove the exosomes in serum. The 3rd - 6th passage of hBMSCs was taken and the supernatant was removed after the cells reached approximately 80% confluence, followed by 2 washes using PBS. The cells were then cultured in the DMEM/F12 supplemented with 10% exosome-free FBS in a CO_2_ incubator at 37 °C for 48 h. The cell supernatant (100 mL) was collected and separated in a 15 mL sterile centrifuge tube. At first, the cell supernatant was successively centrifuged at 300 and then 2000×g for 10 min to remove cell debris and dead cells. Subsequently, cell supernatant was collected and filtered using sterile filter membrane to remove the bacteria, viruses, microorganisms, and macrovesicles. The filtrate was sub-packaged into a 34.5 mL ultrafiltration centrifuge tube. After filling with sterile PBS, the tube was sealed. The cell supernatant was centrifuged at 100,000×g for 70 min and the supernatant was discarded. The precipitate was collected, followed by resuspension in PBS. Finally, cell supernatant was centrifuged at 100,000×g for 70 min, resuspended in PBS, filtered through a 0.22 μm filter membrane, and stored at − 80 °C.

### Nanosight tracking analysis of nanoparticles

A total of 20 μg exosomes were dissolved in 1 mL PBS and vortexed for 1 min so that exosomes were uniformly distributed. Subsequently, the NanoSight Nanoparticle Tracking Analyzer (NTA, Malvern Panalytical, Malvern, UK) was adopted to measure the motion track of each exosome in the screen, which was automatically converted to the diameter and concentration of exosomes according to the Brownian motion principle, and then converted to the original concentration according to the dilution ratio [24].

### Observation of exosomes under the electron microscope

The prepared exosomes were fixed with 4% glutaraldehyde at 4 °C for 2 h, rinsed three times with 0.1 mol/L PBS and fixed in 1% osmium tetraoxide for 2 h. After gradient dehydration of conventional ethanol and acetone, the samples were immersed, embedded, and polymerized in epoxy resin, followed by preparation of 0.5 nm ultrathin sections. After optical location, 60 nm ultrathin sections were prepared, which were then stained with uranium acetate and lead citrate and observed under a JEM-1230 electron microscope (Nihon Denshi, Tokyo, Japan).

### Internalization of exosomes by prostate cancer cells

PKH-26 is a dye specially used for membrane labeling. It can stably bind to the lipid region of exosome lipid membrane and emits red fluorescence, which can be used to track exosomes. Next, 20 μg exosomes were incubated with 1 μL PKH-26 at 37 °C for 15 min, and added with l mL Diluent C and 200 μL l% BSA/PBS. Next, the system was cultured with 3 μL PKH-26 for 20 min at room temperature, and then centrifuged at 100,000×g for 70 min at 4 °C in vacuum, enabling exosomes in the samples to be enriched in 1.13–1.19 g/mL sucrose density and collected. The obtained precipitate was re-suspended in 100 μL l% BSA/PBS. The labeled exosomes were co-cultured with prostate cancer cells. To characterize the transfer of exosomal miR-205, Cy3-labeled miR-205 was initially transfected into hBMSCs. The cells were then washed using PBS and underwent 48 h of incubation with the medium containing exosome-free FBS. The obtained pellet was suspended in serum-free medium to treat LNCaP cells grown on the cover slips, which lasted for 24 h. The LNCaP cells were subsequently fixed with 4% paraformaldehyde and the nuclei were stained with 4,6-diamidino-2-phenylindole (DAPI). The cytoskeleton of LNCaP cells was subjected to selective staining with FITC Phalloidin (YEASEN Biotechnology Co., Ltd., Shanghai, China). At last, the capture of exosomes by prostate cancer cells and exosomal miR-205 was observed under a laser scanning confocal microscope (FV3000, Olympus, Tokyo, Japan).

### Co-culture of exosomes from hBMSCs with LNCaP cells

The exosomes were extracted from the transfected hBMSCs and mixed with LNCaP cells with 60% confluence inoculated in a 24-well plate for 48 h. A total of 20 μg exosomes were added to each well. The cells were then divided into two groups: exo-miR-NC + LNCaP (transduced with exosomes from hBMSCs infected with lentivirus expressing miR-NC and LNCaP cells) and exo-miR-205 + LNCaP (transduced with exosomes from hBMSCs infected with lentivirus expressing miR-205 and LNCaP cells). After incubation, LNCaP cells were washed with PBS for three times, and the subsequent experiments were carried out. The experiment was repeated three times.

### Acetylcholinesterase (ach E) activity assay

The multi-step ultracentrifugation method was used to extract exosomes, which were diluted into 110 μL using PBS. The abovementioned solution (37.5 μL) was added into the 96-well plates, followed by addition of equal volumes of 5,5’dithiobis (2-nitrobenzoic acid) (DTNB) solution (0.1 mmol/L) and thioacetylcholine iodide solution (1.25 mmol/L) to reach a final volume of 300 μL. The optical density (OD) value of each well was measured on a microplate reader at the wavelength of 412 nm after 30 min.

### Nude mouse xenograft model of prostate cancer

A total of 18 male BALB/c nude mice (aged 5 weeks old and weighing 20–22 g) were purchased from Shanghai SLAC Laboratory Animal Co., Ltd. (Shanghai, China). Nude mice were fed in an environment with a constant temperature of 25–27 °C and humidity of 45–50%. The LNCaP single cell suspension (0.2 mL) containing 1 × 10^7^ cells was inoculated subcutaneously to the left armpit of each nude mouse. When the tumor grew to a volume of 100 mm^3^, the nude mice were randomly divided into 3 groups (*n* = 6): PBS, hBMSCs-miR-NC, and hBMSCs-miR-205 (exosomes were extracted from each group and injected with PBS, hBMSCs-miR-NC, and hBMSCs-miR-205, respectively). The hBMSCs were transfected with miR-NC or miR-205 to establish the stably transfected cell lines. Subsequently, a total of 5 × 10^5^ cells were injected into each nude mouse through a tail vein every three days. The tumor size was then measured using a Vernier caliper and the tumor volume (mm^3^) was calculated using the formula: L × W^2^)/2, in which L represented the length of the tumor, W represented the width of the tumor. After treatment for seven times, the mice were euthanized with CO_2_. Thereafter, tumors were removed and weighed. The tumors and other organ samples were frozen with liquid nitrogen or fixed with 4% paraformaldehyde and embedded in paraffin for immunohistochemistry analysis.

### Statistical analysis

Statistical analysis was performed using the SPSS 21.0 software (IBM Corp. Armonk, NY, USA). The measurement data were expressed as mean ± standard deviation. Comparisons of data following normal distribution and homogeneity of variance were tested by paired *t*-test or unpaired *t*-test. The normality test was conducted using the Kolmogorov-Smirnov test. Comparisons of data with normal distribution among multiple groups were analyzed using one-way analysis of variance (ANOVA), followed by Tukey’s post hoc test. The repeated measures ANOVA was used to analyze data of tumor volume at different time points, followed by Bonferroni post hoc test. *p* < 0.05 was considered to be statistically significant.

## Results

### Exosomal miR-205 may affect the progression of prostate cancer by regulating RHPN2

**Fig. 1:**
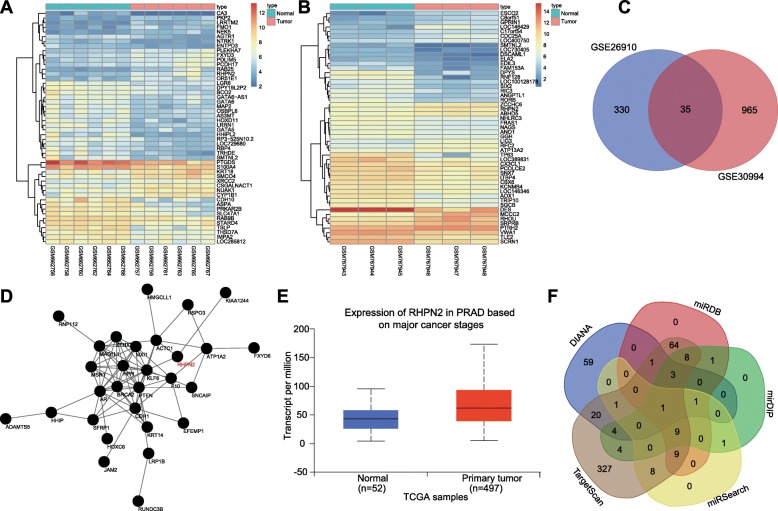
Microarray analysis identifies that exosomal miR-205 may affect the progression of prostate cancer via regulating RHPN2. **a**, **b** heatmaps of prostate cancer-related DEGs in GSE26910 and GSE30994 datasets; the abscissa referred to sample number, and the ordinate referred to gene name; the left dendrogram represents the clustering of gene level; each rectangle corresponds to a sample expression value; the histogram at the upper right referred to color gradation; **c** the intersection of DEGs in prostate cancer-related microarray data GSE26910 and GSE30994; the left circle represented the DEGs in GSE26910, the left circle represented the DEGs in GSE30994, and the middle part represented the intersection of DEGs between them; **d** gene interaction association map of the obtained 35 DEGs and the known genes using MalaCards database; each globule represented one gene, and the connection between the globules represented the interaction between the two genes; **e** RHPN2 expression in TCGA database; the abscissa referred to sample number, and the ordinate referred to gene name; the left box diagram represented RHPN2 expression in normal samples, and the right box diagram represented RHPN2 expression in prostate cancer samples; **f** prediction of miRNAs that may regulate RHPN2; five irregular graphs represented predictions of miRNAs that may regulate RHPN2 in five databases; the middle part represented the intersection of the predicted results among five databases

The prostate cancer-related microarray data (GSE26910 and GSE30994) were obtained from the GEO database. The expression of the genes in GSE26910 and GSE30994 were analyzed and 365 and 3000 DEGs were obtained, respectively. The gene expression heat maps of the top 50 differentially expressed genes (DEGs) in GSE26910 and GSE30994 are shown in Fig. 1a, b. Venn analysis was conducted to define the intersection of the 365 DEGs in GSE26910 and the 1000 DEGs in GSE30994, which identified 35 DEGs in the intersected databases (Fig. 1c). Moreover, the MalaCards database was utilized to retrieve the known genes related to prostate cancer, and the correlation between the obtained DEGs and the known genes was analyzed, and the gene interaction network map was constructed (Fig. 1d). This analysis showed that the RHPN2 gene had significantly elevated expression in prostate cancer samples and interacted with KLF6 gene, which was the core gene in gene interaction network. In addition, it has been demonstrated that RHPN2 plays vital roles in multiple tumors [16, 17], while there were no studies about the effects of RHPN2 on prostate cancer. Through verifying RHPN2 expression in prostate cancer via the TCGA dataset, we found that RHPN2 expression was also upregulated in prostate cancer samples (Fig. 1e). To investigate the upstream regulatory mechanisms of RHPN2, the DIANA, miRDB, mirDIP, miRSearch, and TargetScan databases were employed to predict the miRNAs possibly regulating RHPN2. The findings displayed only one miRNA, miR-205, existed in the intersection (Fig. 1f). Furthermore, a recent study has revealed that BMSCs could secret exosomes carrying miR-205 to exert regulatory functions of miR-205 [25]. Thus, we speculate that exosomal miR-205 might affect the progression of prostate cancer by regulating RHPN2.

### Overexpression of miR-205 inhibits cell proliferation, invasion, and migration, and promotes apoptosis in prostate cancer cells

**Fig. 2:**
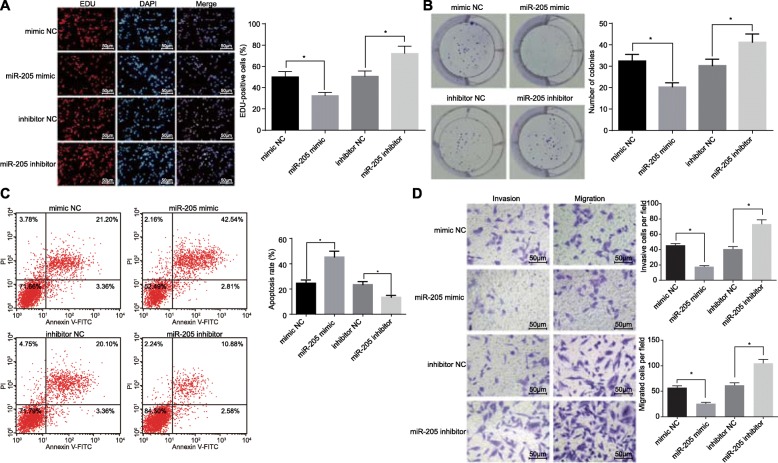
Upregulation of miR-205 suppresses cell proliferation, invasion, and migration, but enhances apoptosis in prostate cancer. **a** proliferation of LNCaP cells after overexpression or inhibition of miR-205 detected using EdU assay (200 ×); **b** colony-forming ability of LNCaP cells after overexpression or inhibition of miR-205 detected using clonogenic assay; **c** apoptosis of LNCaP cells after overexpression or inhibition of miR-205 detected using flow cytometry; Abscissa represents apoptotic cells identified by Annexin V-FITC and ordinate represents dead cells identified by PI; **d** invasion and migration of LNCaP cells after overexpression or inhibition of miR-205 detected using Transwell assay (200 ×); *, *p* < 0.05. Measurement data were expressed as mean ± standard deviation; comparisons between two groups were analyzed using unpaired *t-test*; the experiment was repeated three times independently

To investigate the biological function of miR-205 in tumorigenesis of prostate cancer, we measured proliferation, colony-forming ability, apoptosis, and invasion and migration of LNCaP cells with overexpression of miR-205 or inhibition of miR-205. The LNCap cells transfected with miR-205 mimic showed inhibited proliferation, colony-forming ability, invasion, and migration, as well as enhanced apoptosis compared to the control cells (*p* < 0.05). By contrast, inhibition of miR-205 was shown to reverse the situations in the aforementioned factors induced by overexpressed miR-205 (*p* < 0.05) (Fig. 2a-d). Thus, upregulated miR-205 could inhibit prostate cancer cell proliferation, invasion, and migration, while facilitating apoptosis. However, these effects can be abolished by miR-205 inhibition.

### RHPN2 is a target gene of miR-205

**Fig. 3:**
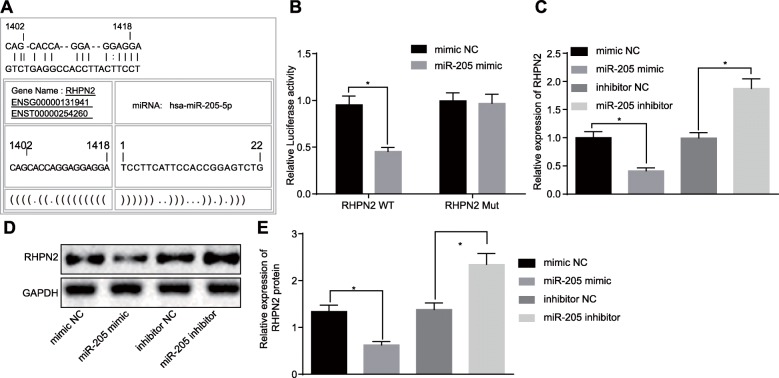
miR-205 targets and negatively regulates RHPN2. **a** predicted binding sites of miR-205 in the 3’UTR of RHPN2; **b** binding of miR-205 to RHPN2 confirmed by dual-luciferase reporter gene assay; **c** RHPN2 mRNA expression in cells after overexpression or inhibition of miR-205 treatment detected by RT-qPCR; **d** RHPN2 protein expression in cells after overexpression or inhibition of miR-205 treatment detected by using western blot analysis; *, *p* < 0.05. Measurement data were expressed as mean ± standard deviation; comparisons between two groups were analyzed using unpaired *t-test*; the experiment was repeated three times independently

To detect the binding site between RHPN2 and miR-205, we used the microRNA.org database (http://www.microrna.org/) and dual-luciferase reporter gene assay. The microRNA.org database displayed a binding site between RHPN2 and miR-205 (Fig. 3a). Additionally, the results of dual-luciferase reporter gene assay showed that luciferase activity of RHPN2 3’UTR WT was significantly inhibited by miR-205 mimic (*p* < 0.05), but no difference was found in that of RHPN2 3’UTR MUT (*p* > 0.05) (Fig. 3b). Moreover, in prostate cancer cells, RHPN2 mRNA and protein expression were reduced upon miR-205 mimic treatment, while miR-205 inhibition led to increased RHPN2 mRNA and protein expression (Fig. 3c-e). All these findings reveal that RHPN2 is a target gene of miR-205, which negatively regulates RHPN2.

### Silencing of RHPN2 inhibits cell proliferation, invasion, and migration, and promotes apoptosis of prostate cancer cells

**Fig. 4:**
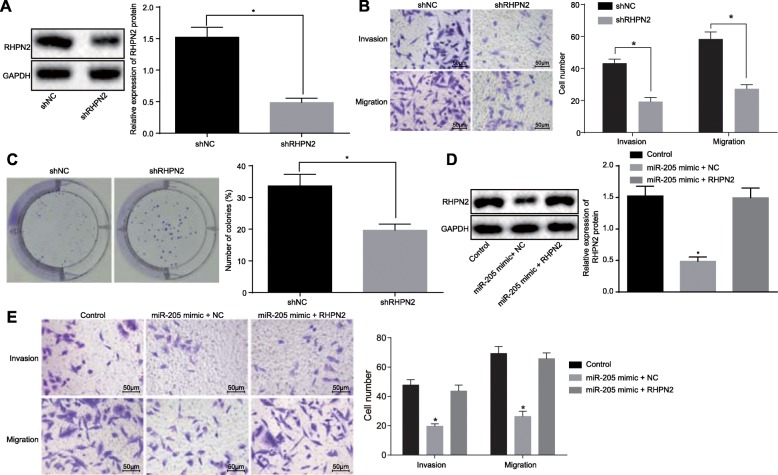
Knockdown of RHPN2 suppresses prostate cancer cell proliferation, invasion, and migration while accelerates apoptosis. **a** RHPN2 protein expression in cells after silencing of RHPN2 determined using western blot analysis; *, *p* < 0.05. **b** prostate cancer cell invasion and migration upon treatment with shRHPN2 plasmids detected using Transwell assay (200 ×); *, *p* < 0.05. **c** prostate cancer cell colony-forming ability upon treatment with shRHPN2 detected using clonogenic assay; *, *p* < 0.05. **d** RHPN2 protein expression in cells treated with upregulated miR-205 or upregulated RHPN2 detected using western blot analysis; **e** prostate cancer cell invasion and migration following treatment by upregulated miR-205 or upregulated RHPN2 detected using Transwell assay (200 ×); *, *p* < 0.05 vs. the control group. Measurement data were expressed as mean ± standard deviation; comparisons between two groups were analyzed using unpaired *t*-test; comparisons among multiple groups were analyzed by one-way analysis of variance; the experiment was repeated three times independently

To elucidate the effects of RHPN2 on prostate cancer, the prostate cancer cell invasion and migration were detected with or without RHPN2 silencing. The silencing efficiency was first verified via immunoblotting (Fig. 4a). The Transwell assay displayed that silencing RHPN2 obviously suppressed prostate cancer cell invasion and migration (*p* < 0.05) (Fig. 4b). The clonogenic assay subsequently showed that downregulated RHPN2 significantly repressed the colony-forming ability of prostate cancer cells (*p* < 0.05) (Fig. 4c). Furthermore, exogenous overexpression of RHPN2 could rescue miR-205-induced suppression of prostate cancer cell invasion and migration (*p* < 0.05) (Fig. 4d-e). Thus, RHPN2 knockdown suppressed proliferation, invasion, and migration, and promoted apoptosis of prostate cancer cells.

### Characterization of hBMSCs-secreted exosomes

**Fig. 5:**
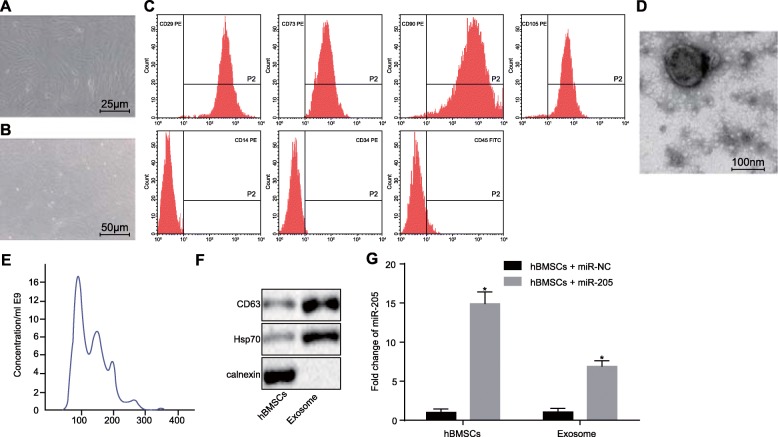
Characterization of hBMSCs-secreted exosomes. **a**, **b** morphological characteristics of hBMSCs at 24 h (× 400) and day 8 of culture (× 200); **c** characterization of the surface makers of hBMSCs by flow cytometry; **d** ultrastructure of exosomes observed under a transmission electron microscope (scale bar = 100 nm); **e** analysis of size and concentration of exosomes by nanoparticle tracking analyzer; **f** exosome surface markers (Hsp70 and CD63) and exosome negative marker CANX detected using western blot analysis; **g** miR-205 expression in hBMSCs and exosomes determined using RT-qPCR; *, *p* < 0.05. Measurement data were expressed as mean ± standard deviation; comparisons between two groups were analyzed using unpaired *t*-test; the experiment was repeated 3 times independently

Observation of adherent hBMSCs cultured with the DMEM/F12 supplemented with 10% FBS under the inverted microscope showed that they were large, polygonal, spindle-like, or spindle-shaped adherent cells with large nuclei in the middle of the cell. The hBMSCs grew in clusters with an uneven whirlpool distribution. After incubation for 8 days, the cell confluence reached 80–90% (Fig. 5a, b). Flow cytometry indicated that CD29 (98%), CD73 (97%), CD90 (96%), and CD105 (95%) were highly expressed, while CD14 (0.22%), CD34 (0.83%), and CD45 (2.11%) were barely expressed (Fig. 5c). Transmission electron microscope examination of microvesicle samples showed that the exosomes had characteristic morphological characteristics, ranging in diameter from 40 to 100 nm, being round or dish-shaped in shape, containing low-density substances and complete lipid membrane vesicles (Fig. 5d). Subsequently, the Nanosight NS300 nanoparticle tracking analyzer showed that the exosomes had a mean size of 98.5 ± 0.8 and concentration of 5.34 × 10^10^ ± 1.85 × 10^9^/mL (Fig. 5e). Furthermore, western blot analysis showed that the exosomes secreted by hBMSCs expressed the exosome-specific markers Hsp70 and CD63 but did not express the exosome negative marker CANX (Fig. 5f), indicating that hBMSCs could indeed secrete exosomes. Next, we introduced miR-205 mimic in hBMSCs and detected the expression of miR-205 in hBMSCs-derived exosomes and found that exosomes derived from miR-205 mimic-treated hBMSCs also showed high expression of miR-205 compared with the control group (Fig. 5g).

### Exosomal miR-205 derived from hBMSCs inhibits RHPN2 expression in LNCaP cells

**Fig. 6:**
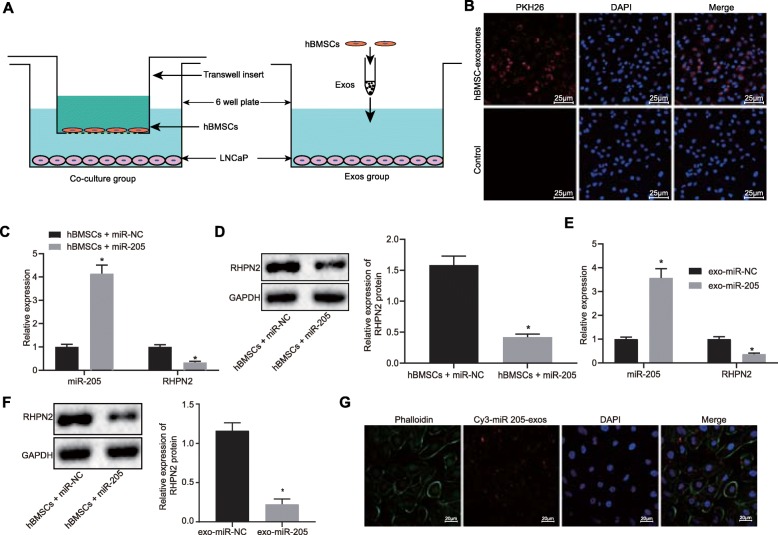
hBMSCs-derived exosomal miR-205 suppresses RHPN2 expression in LNCaP cells. **a** co-culture pattern of hBMSCs or hBMSC-derived exosomes with LNCaP cells; **b** the internalization of exosomes by prostate cancer cells observed under a confocal microscope (× 400); **c-d** RHPN2 expression after co-culture of hBMSCs with LNCaP cells determined using RT-qPCR and western blot analysis; **e-f** RHPN2 expression after co-culture of exosomes from hBMSCs with LNCaP cells determined using RT-qPCR and western blot analysis; **g** the binding of hBMSCs-derived exosomes with Cy3-labeled red fluorescence to LNCaP cells observed under a confocal microscope; exosomes carrying Cy3-labeled miR-205 were red, nuclei stained with DAPI were blue; cells labeled with phalloidin were stained green. Scale bar = 20 μm; *, *p* < 0.05. Measurement data were expressed as mean ± standard deviation; comparisons between two groups were analyzed using unpaired *t*-test; the experiment was repeated three times independently

Next, to observe whether prostate cancer cells affected the internalization of exosomes derived from hBMSCs, we co-cultured hBMSCs or their isolated exosomes with LNCaP cells, respectively (Fig. 6a). As shown in Fig. 6b, hBMSCs-derived exosomes following co-culture with LNCaP cells accumulated on the surface of prostate cancer cell membrane, and prostate cancer cells could produce exosomes, while there was no marked exosome attachment on the surface of prostate cancer cell membrane in the blank control. In order to further investigate the roles of hBMSCs in LNCaP cells, the hBMSCs were co-cultured with LNCaP cells. To determine whether in vitro transfer of miR-205 could effectively inhibit endogenous RHPN2 in tumor cells, RT-qPCR and western blot analysis were performed to measure the mRNA and protein expression of RHPN2 in LNCaP cells co-cultured with hBMSCs-miR-205 (Fig. 6c, d). The results showed that hBMSCs could transfer miR-205 to LNCaP cells, thus reducing the mRNA and protein expression of RHPN2 in LNCaP cells. Furthermore, RT-qPCR and western blot analysis also showed (Fig. 6e-f) that exosomal miR-205 could be transferred to LNCaP cells and consequently inhibited the mRNA and protein expression of RHPN2 in LNCaP cells. Concurrently, we used red fluorescence Cy3-labeled miR-205 to transfect the hBMSCs (Fig. 6g). After 48 h, we collected the supernatant of the cells for ultracentrifugation to obtain the exosomes, which were then co-cultured with LNCaP cells for 12 h, fixed, stained and photographed. There was Cy3-labeled red fluorescence in LNCaP cells, which indicated that Cy3-labeled miR-205 was able to effectively transmit to LNCaP cells through exosomes. The aforementioned data suggested that hBMSCs could deliver miR-205 to LNCaP cells via exosomes, thereby inhibiting RHPN2 expression in LNCaP cells.

### GW4869 inhibits secretion of exosomes from hBMSCs

**Fig. 7:**
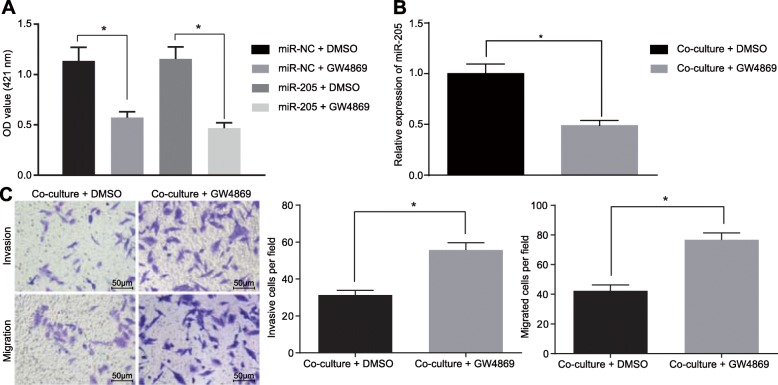
GW4869 suppresses the production of exosomes from hBMSCs, thus reducing the delivered exosomal miR-205. **a** release of exosomes from hBMSCs in cells treated with GW4869 or DMSO detected using Ach E activity assay; **b** miR-205 expression in LNCaP cells treated with MSC medium following or without GW4869 treatment as measured using RT-qPCR; **c** migration and invasion of LNCaP cells treated with MSC medium following or without GW4869 treatment as measured using Transwell assay (× 200). *, *p* < 0.05. Measurement data were expressed as mean ± standard deviation; comparisons between two groups were analyzed using unpaired *t*-test; the experiment was repeated three times independently

Having investigated the roles of exosomal miR-205 from hBMSCs in RHPN2 expression in LNCaP cells, we next used GW4869, an exosome inhibitor, to treat hBMSCs with the aim to elucidate whether miR-205 was delivered to LNCaP cells via exosomes and altered their biological functions. First, we added an exosome inhibitor GW4869 or DMSO to the hBMSC medium, respectively. Ach E activity assay showed that Ach E activity was reduced in cells treated with miR-NC + GW4869 and miR-205 + GW4869 in comparison to treatment with miR-NC + DMSO or miR-205 + DMSO, suggesting the decreased release of exosomes (*p* < 0.05) (Fig. 7a). Subsequently, LNCaP cells were further treated with MSC medium overexpressing miR-205 with or without GW4869 treatment. The findings displayed that the expression of miR-205 in LNCaP cells was decreased (Fig. 7b), while migration and invasion of LNCaP cells were promoted upon treatment with MSC medium overexpressing miR-205 following GW4869 treatment (*p* < 0.05) (Fig. 7c). Thus, GW4869 could effectively suppress the production of exosomes from hBMSCs and then affect the transfer of miR-205 in hBMSCs to prostate cancer cells, suggesting that hBMSCs impact the biological functions of prostate cancer cells via exosomes.

### Exosomal miR-205 derived from hBMSCs inhibits LNCaP cell proliferation, invasion, and migration, and enhances apoptosis

**Fig. 8:**
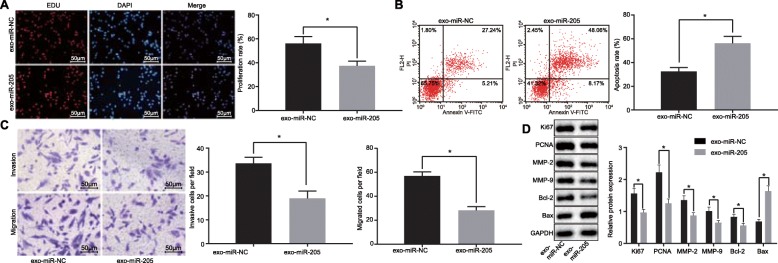
hBMSCs-derived exosomal miR-205 inhibits LNCaP cell proliferation, invasion, and migration and enhances apoptosis. **a** proliferation of LNCaP cells treated with hBMSCs and miR-205 measured using EdU assay (× 200); **b** apoptosis of LNCaP cells treated with hBMSCs and miR-205 measured using flow cytometry; Abscissa represents apoptotic cells identified by Annexin V-FITC and ordinate represents dead cells identified by PI; **c** invasion and migration of LNCaP cells treated with hBMSCs and miR-205 measured using Transwell assay (× 200); **d** protein band patterns and levels of Ki67, PCNA, MMP-2, MMP-9, Bcl-2, and Bax measured in LNCaP cells treated with hBMSCs and miR-205 determined using western blot analysis; *, *p* < 0.05. Measurement data were expressed as mean ± standard deviation; comparisons between two groups were analyzed using unpaired *t*-test; the experiment was repeated 3 times independently

Next, we tested the effects of miR-205 secreted from hBMSCs on LNCaP cell functions. As shown in Fig. 8a-c, hBMSCs-derived miR-205 repressed proliferation, invasion, and migration and enhanced apoptosis of LNCaP cells co-cultured with hBMSCs-derived exosomes (*p* < 0.05). Next, western blot analysis proliferation-related factors (Ki67 and PCNA), invasion-related factors (MMP-2 and MMP-9), and apoptosis-related factors (Bcl-2 and Bax), which revealed that hBMSCs-derived miR-205 inhibited reduced protein levels of Ki67, PCNA, MMP-2, MMP-9, and Bcl-2 and elevated Bax protein level (all *p* < 0.05) (Fig. 8d). Thus, miR-205 can be transferred to LNCaP cells by hBMSCs via exosomes and then suppresses LNCaP cell proliferation, invasion, and migration in addition to facilitating apoptosis.

### miR-205 secreted from hBMSCs suppresses tumor growth in vivo by downregulating RHPN2

**Fig. 9:**
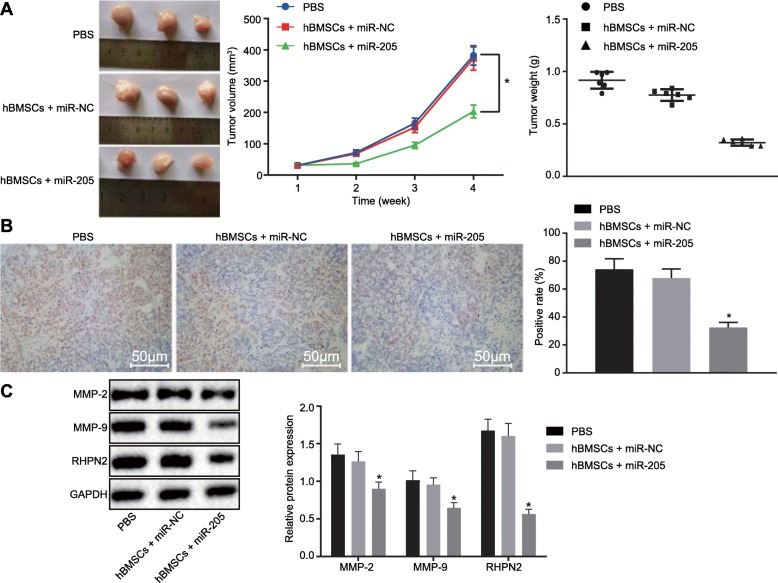
hBMSCs-derived exosomal miR-205 delays tumor growth in vivo by inhibiting RHPN2. **a** tumor size and volume of nude mice; **b** RHPN2 expression in mice detected using immunohistochemistry (× 200); **c** protein levels of MMP-2, MMP-9, and RHPN2 in mice determined using western blot analysis; *, *p* < 0.05, vs. nude mice treated with exosomes from hBMSCs-miR-NC or PBS. Measurement data were expressed as mean ± standard deviation; comparisons among multiple groups were analyzed by one-way analysis of variance; comparisons of tumor volume at different time points were analyzed by repeated measures analysis of variance; *n* = 6

We next explore the role of hBMSCs-derived miR-205 in LNCaP tumor growth in vivo. Tumor xenograft nude mouse models were injected with hBMSCs-miR-205, hBMSCs-miR-NC, and PBS via tail vein to investigate the inhibitory role of miR-205 in prostate cancer in vivo. The tumor volume and weight were measured and tumor tissues were stained with immunohistochemistry. As depicted in Fig. 9a, hBMSCs-derived miR-205 reduced tumor volume and weight (*p* < 0.05). Additionally, the results of immunohistochemistry indicated that hBMSCs-derived miR-205 significantly decreased the RHPN2 level in prostate cancer tissues (*p* < 0.05) (Fig. 9b). Furthermore, western blot analysis displayed that hBMSCs-derived miR-205 reduced the protein levels of MMP-2, MMP-9, and RHPN2 (*p* < 0.05) (Fig. 9c). These results indicate that hBMSCs-derived miR-205 inhibits tumor growth in vivo by downregulating RHPN2.

## Discussion

One of the standard biomarkers for its diagnosis is prostate-specific antigen, which has its limitations, amplifying the risk of over-diagnosis and harmful overtreatment for prostate cancer [26]. In recent years, exosomes derived from stromal cells have been suggested to serve as mediators in human cancers [27]. Our study aimed to explore the effect of exosomes derived from hBMSCs overexpressing miR-205 on the progression of prostate cancer in vitro and in vivo. Collectively, our results suggest that exosome-mediated transfer of miR-205 from hBMSCs to prostate cancer cells suppresses proliferation, migration, and invasion of prostate cancer cells via downregulation of RHPN2, ultimately attenuating progression of prostate cancer.

Initially, our initial results demonstrated that miR-205 was downregulated while RHPN2 was upregulated in prostate cancer cells. As shown previously, the abnormal expression of miRNAs participates in a variety of malignancies, including prostate cancer [13]. Several miRNAs, such as miR-29b and miR-34b, are reported to be poorly expressed in prostate cancer tissues [28, 29]. It has also been demonstrated that miR-205 displays reduced expression in prostate cancer tissues [30], which was in line with our study. A previous study has confirmed that increased expression of RHPN2 in human glioma samples [18]. However, there was no research focusing on its expression in prostate cancer. Moreover, the bioinformatics analysis in the present study displayed that RHPN2 was a target of miR-205, which negatively regulated RHPN2 by binding to the 3’UTR of RHPN2. RHPN2 has been reported to be targeted by miR-182-5p, miR-363-3p, miR-200a-3p and miR-141-3p in ovarian endometriosis [31].

Our present study implies that hBMSCs can transfer miR-205 to LNCaP cells through exosomes. Cancer cells have been shown to release a variety of exosomes that can deliver miRNAs to metastatic loci prior to the arrival of the cancer cells, stimulating alterations of the cancer microenvironment and thereby promoting tumor metastasis [32]. Recent evidence has documented that exosomes containing miRNAs serve as a biomarker for prostate cancer [14]. Moreover, MSC-derived exosomes carrying miR-205 have a regulatory function in wound healing [25]. These findings support that hBMSCs can deliver miR-205 to LNCaP cells through exosomes.

Furthermore, both in vitro and in vivo experimental results in the present study revealed that miR-205 secreted from hBMSCs inhibited LNCaP cell proliferation, invasion, and migration, tumor growth, and promoted apoptosis by downregulating its target RHPN2. Previous evidence revealed that miRNAs were closely associated with the tumorigenesis and progression of prostate cancers [12]. For instance, Wang et al. have confirmed that restoration of miR-205 could suppress prostate cancer cell growth, colony formation, migration, invasion, as well as induce cell apoptosis in vitro and inhibits tumor formation in vivo [33]. Previously, MSCs can secrete exosomes containing miR-205, which then plays a regulatory role in the skin wound healing [25]. Upregulation of exosomal miR-1246 is also demonstrated to contribute to the inhibition of cell proliferation, invasion, and migration in vitro and xenograft tumor growth in vivo and facilitation of apoptosis in prostate cancer [34]. Moreover, exosomes released from MSCs play a vital role in angiogenesis of endothelial cells by transferring miR-125a [35]. BMSCs are known to differentiate into a variety of mesodermal lineages, such as osteoblasts and chondrocytes, and the ability of BMSCs to home to sites of tumors has attracted great interests of these cells as potential therapeutic targets [8]. Furthermore, RHPN2 triggers the progression of malignant glioma via activation of RhoA [17], indicating that silencing of RHPN2 exerts an inhibitory impact on the development of malignancy. All these findings consistently indicated that hBMSCs-derived exosomal miR-205 possessed a great therapeutic potential in prostate cancer.

## Conclusion

**Fig. 10:**
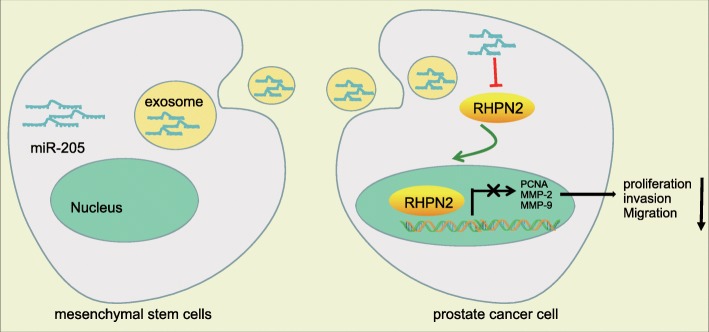
Mechanistic investigations indicated that exosomes secreted from hBMSCs could transfer miR-205 to prostate cancer cells and consequently repress prostate cancer cell proliferation, invasion, and migration whereas potentiating apoptosis by inhibiting RHPN2

In conclusion, hBMSCs-derived exosomal miR-205 could potentially be transferred to prostate cancer cells, thus inhibiting proliferation, invasion, and migration of prostate cancer cells, and promoting their apoptosis via suppression of RHPN2 (Fig. 10). Thus, elevation of miR-205 may be a new strategy for treating prostate cancer, although much further research is required to translate this model into the clinic.

## Data Availability

The datasets generated/analyzed during the current study are available.

## Abbreviations

3’UTR: 3’untranslated region
Ach E: Acetylcholinesterase
ANOVA: Analysis of variance
Bax: Bcl-2-associated X protein
BCA: Bicinchoninic acid
Bcl-2: B-cell lymphoma 2
BMSCs: Bone marrow-derived mesenchymal stem cells; Rho: Ras-homologous
CANX: Calnexin
cDNA: complementary DNA
DAPI: 4,6-diamidino-2-phenylindole
DEGs: Differentially expressed genes
DEPC: Diethylpyrocarbonate
DMEM: Dulbecco’s modified Eagle’s medium
DTNB: 5,5’dithiobis (2-nitrobenzoic acid)
EDTA: Ethylenediaminetetraacetic acid
EdU: 5-ethynyl-2′-deoxyuridine
F12: Ham’s F-12 medium
FBS: Fetal bovine serum
FITC: Fluorescein isothiocyanate
GAPDH: Glyceraldehyde-3-phosphate dehydrogenase
GEO: Gene Expression Omnibus
hBMSCs: Human bone marrow mesenchymal stem cells
hMSCs: Human mesenchymal stem cells
miRNAs: MicroRNAs
MMP: Matrix metalloproteinase
MSCs: Mesenchymal stem cells
MUT: Mutant
NC: Negative control; sh: short hairpin RNA
OD: Optical density
PBS: Phosphate-buffered saline
PBST: PBS containing 0.1% Tween-20
PCNA: Proliferating cell nuclear antigen
PI: Propidium iodide
PVDF: Polyvinylidene fluoride
RPMI: Roswell Park Memorial Institute
RT-qPCR: Reverse transcription quantitative polymerase chain reaction
SDS-PAGE: Sodium dodecyl sulfate polyacrylamide gel electrophoresis
TCGA: The Cancer Genome Atlas
WT: Wild type
