# Correction: Human bone marrow mesenchymal stem cells-derived microRNA-205-containing exosomes impede the progression of prostate cancer through suppression of RHPN2

**DOI:** 10.1186/s13046-022-02417-y

**Published:** 2022-06-18

**Authors:** Shuangjian Jiang, Chengqiang Mo, Shengjie Guo, Jintao Zhuang, Bin Huang, Xiaopeng Mao

**Affiliations:** 1 Department of Urology Surgery, the First Affiliated Hospital, Sun Yat-Sen University, No. 58, Zhongshan No. 2 Road, Guangzhou, 510080 Guangdong Province People’s Republic of China; 2 Department of Urology Surgery, Sun Yat-sen University Cancer Center, Guangzhou, 510060 People’s Republic of China; 3 Department of Urology Surgery, the Eastern Hospital of the First Affiliated Hospital, Sun Yat-Sen University, Guangzhou, 510700 People’s Republic of China

**Correction: J Exp Clin Cancer Res 38, 495 (2019)**

**https://doi.org/10.1186/s13046-019-1488-1**

Following publication of the original article [1], an error was identified in Fig. 8; specifically:

- Fig. 8C: Incorrect image used for migration experiment of exo-miR-NC (bottom left image); correct image is now used.

The corrected figure is given here. The correction does not have any effect on the final conclusions of the paper.

**Fig. 8:**
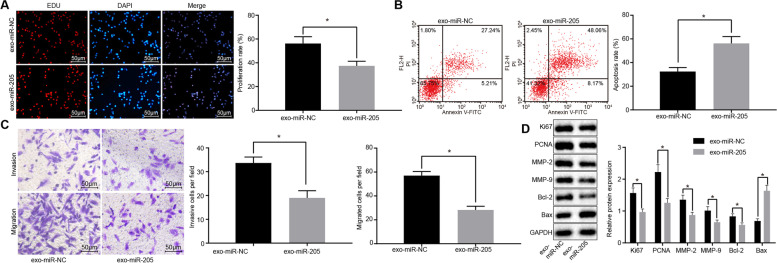
hBMSCs-derived exosomal miR-205 inhibits LNCaP cell proliferation, invasion, and migration and enhances apoptosis. **a** proliferation of LNCaP cells treated with hBMSCs and miR-205 measured using EdU assay (× 200); **b** apoptosis of LNCaP cells treated with hBMSCs and miR-205 measured using flow cytometry; Abscissa represents apoptotic cells identified by Annexin V-FITC and ordinate represents dead cells identified by PI; **c** invasion and migration of LNCaP cells treated with hBMSCs and miR-205 measured using Transwell assay (× 200); **d** protein band patterns and levels of Ki67, PCNA, MMP-2, MMP-9, Bcl-2, and Bax measured in LNCaP cells treated with hBMSCs and miR-205 determined using western blot analysis; *, *p* < 0.05. Measurement data were expressed as mean ± standard deviation; comparisons between two groups were analyzed using unpaired *t*-test; the experiment was repeated 3 times independently
